# Accurate and Reliable Assessment of Heart Rate in Real-Life Clinical Settings Using an Imaging Photoplethysmography

**DOI:** 10.3390/jcm11206101

**Published:** 2022-10-17

**Authors:** Edem Allado, Mathias Poussel, Anthony Moussu, Oriane Hily, Margaux Temperelli, Asma Cherifi, Veronique Saunier, Yohann Bernard, Eliane Albuisson, Bruno Chenuel

**Affiliations:** 1 CHRU-Nancy, Exploration Fonctionnelle Respiratoire—Centre Universitaire de Médecine du Sport et Activités Physiques Adaptées, F-54000 Nancy, France; 2 DevAH, Université de Lorraine, F-54000 Nancy, France; 3 OMEOS, F-54000 Nancy, France; 4 CHRU-Nancy, Direction de la Recherche Clinique et de l’Innovation, F-54000 Nancy, France; 5 CNRS, IECL, Université de Lorraine, F-54000 Nancy, France; 6 Département du Grand Est de Recherche en Soins Primaires: DEGERESP, Université de Lorraine, F-54000 Nancy, France

**Keywords:** heart rate, remote photoplethysmography, vital signs

## Abstract

Remote photoplethysmography imaging (rPPGc) is a new method measuring essential parameters, such as heart rate (HR), which uses a video camera during teleconsultation. Our work aimed to evaluate the accuracy of such remote measurements compared with existing contact point measurement methods in real-life clinical settings. The prospective hospital-based study recruited 1045 patients who required a pulmonary function test. For each patient, measurements of HR using a standard electrocardiogram acquisition system (gold standard) were carried out concomitantly with the measurements made by the rPPGc system (Caducy v1.0.0) taken within a 60 s timeframe. Age, gender, and skin phototype were collected. We performed an intraclass coefficient correlation (ICC) and Bland–Altman plotting to determine the accuracy and precision of the rPPGc algorithm readings. We achieved measurement of HR using the two methods in 963 patients. The ICC measured at a 60 s timeframe, and when we compared the rPPGc with the gold standard, it had a 95% confidence interval (CI95) value of 0.886 [0.871:0.899]. In all, 94.6% (*n* = 911) patients showed promising results with a CI95 in Bland–Altman plotting. Fifty-two measurements were discordant, and further analysis established the method’s accuracy at 96.2%. Our results described a good accuracy and correlation between the rPPGc system and the gold standard, thus paving the way for more precise care via telemedicine.

## 1. Introduction

Since the beginning of the COVID-19 pandemic, the healthcare system has been under tremendous pressure to continue to provide good care for patients while minimising exposure to infections and protecting both health workers and patients [1]. In this particular context, telemedicine and mobile health technologies have been suggested as an alternative to conventional medicine. At present, such technologies are disadvantageous since they do not provide access to essential health parameters such as heart rate (HR). Innovative technologies are currently being developed to overcome this limitation. In addition to their use in telemedicine, the development of such solutions, using affordable and accessible tools, could be a valuable resource for healthcare consultations in low-income countries [2]. If teleconsultations are to produce remote conditions that approach those in face-to-face interactions, such medical interpretation (diagnosis, prognosis, treatment) requires high-quality data. Accurate remote assessments of relevant physiological variables, independent of instruments that the patients manage themselves, metrologically validated, and useful in real-life settings, are therefore fundamental preconditions for analysis and for guaranteeing the clinical value of the data.

Developed in 1937, photoplethysmography (PPG) is a noninvasive optical technique that can detect microvascular blood volume changes in tissues. PPG techniques raised the possibility of measuring HR and oxygen saturation using the absorption of light by the blood [3,4]. In the last 20 years, a modernised version of this technique (rPPG) has been developed to measure those parameters in a remote fashion [5,6,7]. rPPG is a noncontact, video-based method relying on blood absorption of light. The algorithm measures subtle changes in blood volume by capturing pixel intensity changes from the skin, particularly in the green channel. In fact, previous studies have shown that the green channel has the best signal-to-noise ratio [8,9,10].

Several studies have used rPPG for measuring vital signs in laboratory settings in groups of up to over 1300 subjects [11]. However, very few studies have been carried out in the clinical setting, and even fewer on such a large sample of patients as mentioned by Pham et al. [9,12,13,14].

To test whether pathological episodes can be distinguished from artifacts, we addressed the clinical relevance of the rPPG technique in subjects for whom physiological parameter measurements are crucial. 

Indeed, to be able to use this new tool in current practice, it is essential to study the limits of rPPG in real-life clinical settings, among a large sample, including patients with various stable diseases and elderlies.

In order to assess the reliability of HR measurements by rPPG compared to a standard acquisition system (gold standard), we designed a clinical trial with a large cohort of adult patients (both out- and inpatients with stable clinical status).

## 2. Materials and Methods

This interventional monocentric study was performed at a French hospital (University Hospital of Nancy) between December 2020 and May 2021. A total of 1045 adult patients managed in the Respiratory Function Exploration and Sports Medicine Department and who required a pulmonary function test were included in the study. Patients aged over 18 years, with the ability to perform a pulmonary function test, and with a stable clinical status were included in the study. The exclusion criteria were pregnant women or women of childbearing potential without effective contraception. The complete protocol and method has been described previously [15].

Patients underwent a specific physical examination to collect gender, age, body mass index (BMI), Fitzpatrick skin colour scale phototype (FSP), patient history, and resting HR [16]. Patients were at rest and comfortably seated on a chair in front of a computer using a webcam and rPPG system. HR (unit: heart beats per minute (bpm)) measurements using the experimental system rPPGc (remote photoplethysmography imaging Caducy V1.0.0, I-Virtual, Metz, France) and the standard acquisition electrocardiogram (ECG) system (gold standard) were performed simultaneously (PowerLab Acquisition Station, AdInstruments, Dunedin, New Zealand). Videos were acquired at 30 FPS with the webcam of an ASUS laptop model:X512J, Intel^®^ I5 1.00 Ghz. Patients were seated at about 70–100 cm away from the camera. Measurements were acquired in ambient light and ordinary conditions. PPG signals were extracted from the forehead region. To carry out this study it is essential to synchronize both the rPPG and the ECG systems on the same time base. To do so, we used the Powerlab system. Since this system is commonly used in laboratory setting, we performed a concordance analysis between the Powerlab system and a medical device (MASIMO Radical.7) on 20 subjects for 60 s. The modal measurement visualized by the practitioner was noted as well as that given by the Powerlab system. The concordance was excellent: ICC: 0.995 CI95 [0.990–0.998].

Readings and recordings were taken at three different timeframes: 30, 60, and 120 s. The heart rate was assessed by counting the number of heart beats on different time bases during recordings of 120 s. The first 30 s were considered for the so-called 30s recordings, and the first 60 s for the so-called 60s.

To analyse HR, descriptive analyses were conducted according to the nature and the distribution of the variable. Qualitative variables were described with frequencies and percentages; quantitative variables were reported as mean ± standard deviation (SD). The intraclass correlation coefficient (ICC) with a 95% confidence interval (CI) was used to measure the concordance between the two measurement systems. For interpreting ICC, values lower than 0.5, between 0.5 and 0.75, between 0.75 and 0.9, and greater than 0.90 were used as being indicative of poor, moderate, good, and excellent reliability, respectively [17]. 

A Bland–Altman plot was applied to analyse the accuracy between the two measurement systems at 60 s (rPPGc and gold standard). Analyses were performed using IBM SPSS Statistics V.23, and *p* values < 0.05 were considered statistically significant.

This study received approval from the French Ethics Committee (CPP TOURS—Région Centre—Ouest 1—2020T1-30 DM at 27 October 2020) and from the French Agency for the Safety of Health Products (ANSM registration n°.I-RCB 2020-A02428-31). It was conducted according to the European Good Clinical Practice (GCP) recommendations, the general ethical principles of the Declaration of Helsinki, and specific French regulations. Before giving their written consent to participate in the trial inclusion, patients received, both verbally and in written form, a full description of the study’s objectives, its progress, and its constraints. The study protocol is registered at http://www.clinicaltrials.gov (ClinicalTrials.gov ID: NCT04660318—date of first registration, 9 December 2020).

## 3. Results

**Figure 1:**
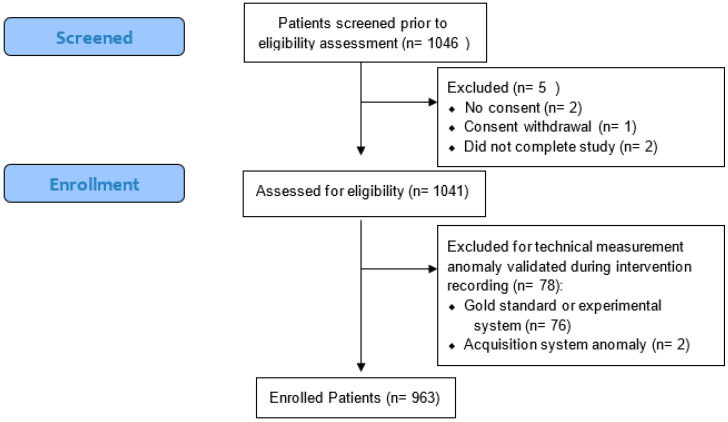
Flow chart of patients.

Of the 1046 patients eligible to take part in the study, 963 were enrolled in the final analysis as shown in Figure 1. Of these, 51.1% were men with an average age of 56.6 (16.0) years and with a range of 74 [min: 18–max: 92] (Table 1).

**Figure 2:**
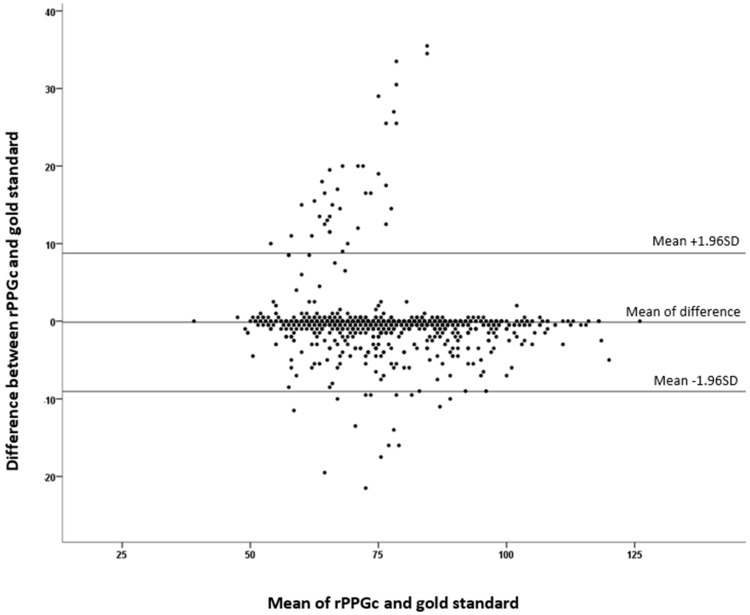
Bland–Altman plot showing the agreement between rPPGc and the control at 60 s.

Comparison of the two techniques showed an ICC of 0.886 CI95 [0.871–0.899] at 60 s with an excellent concordance, and 94.6% of patients were in CI95 in Bland–Altman plotting (Figure 2). We observed 52 measurements falling out of this range; further analysis pointed up 37 (corresponding to 3.8%) measurement errors originating from the rPPGc system, compared to the gold standard method, which was the source of 1.6% errors (*n* = 15).

Criteria such as age and gender had no effect on measurement precision. In fact, the average ICC for women was 0.858, with 95% being in the CI [0.830:0.881], whilst that for men was 0.908 with 95% being in the CI [0.891:0.923]. Considering both genders, the ICC was always higher than 0.80 for patients aged between 18 and 92 years (Table 2). 

Patients with moderate-risk obesity (class 2) showed reduced measurement accuracy as demonstrated in Table 3, but this difference was not seen in those with high-risk obesity (class 3). 

When considering different skin phototypes, results showed a high level of accuracy except for the two darkest skin tones (FSP 5 and 6), for which there were too few patients (*n* = 8 and *n* = 5, respectively) to consider data as significant. From patients with dermatological diseases, comparison of the two techniques showed an ICC of 0.933 CI95 [0.866–0.961] at 60 s. 

The analysis performed on the entire population has shown a close to perfect correlation between the two measurement systems under consideration for measurements at 30, 60, and 120 s timeframes (Table 4). 

## 4. Discussion

The study described good reliability between the gold standard and the rPPGc techniques, with excellent agreement using Bland–Altman plotting of the two systems. The concordance analysis between patients of the same group was close to perfect, showing that a 30 s timeframe measure was sufficient to obtain a satisfactory HR correlation. 

This thorough clinical trial has shown results as encouraging as those observed in laboratory conditions [18,19]. They pave the way for more general use by nonexperts and on a restricted time scale. The Bland–Altman Plot results show a bias in measure (mean difference) to 0 with good general agreement; however, the confidence interval is CI95 [−18.09–17.53]. The data distribution being normal, the confidence interval is influenced by the standard deviation, and therefore by the maximum (71) and minimum (−43) values of the data. It is also observed that the dispersion is more important for the HR values ranging from 50 to 90 bpm. However, this variability can be explained by the fact that 84% of the study population have results in this range.

The differences observed between the estimated and the actual physiological signals could be for multiple reasons Firstly, rPPGc is a noncontact video-based method measuring pulse rate by the change in blood volume by capturing pixel intensity changes from the skin to measure pulse rate [9]. In contrast, ECG-derived HR data provide direct access to heart rhythm by measuring the electrical activity of the heart. In this gold standard setting, measurement errors are mainly due to the way the patches are placed [20,21]. 

In the very particular context of the COVID-19 pandemic, the future prospect for the use of PPG remote assessment of HR, which permits an adequate distance from the patient, appears very attractive.

A limited number of studies have been performed outside of the laboratory setting and that consider patients with such a large diversity of age, gender, or skin colour, which could all potentially bring measurement discrepancies [12,22]. In fact, our results have demonstrated no significant effect of BMI, gender, or age on the accuracy of rPPG-derived HR data. Additionally, although the measurements are made on the face, where adipocyte content decreases with age and is often covered with make-up, none of those parameters affected the precision of the measurement. The higher ICC value in patients with dermatological issues was seen, suggesting dermatological alterations do not affect measurement precision.

As seen in the literature, skin tone as an important source of crucial bias remains controversial. In a study provided by Bent et al. on the sources of inaccuracy in wearable optical HR sensors, no statistically significant difference across skin tones was observed [23]. However, as the amount of melanin present in darker skin tones is higher, it could absorb a significant amount of incident light and thus degrade the quality of the camera-based PPG signal, making the system ineffective for extracting vital signs in darker skin tones [23]. More recently, in 2019, Dasari et al. demonstrated that excess subject movement and rapid lighting changes cause highest error level and that skin tones may not be a critical factor impacting the bias in estimating pulse rate [24]. In our study, we have demonstrated a very robust HR concordance compared to ECG trace among skin tone groups 1 to 4 using the FSP. For patients of FSP 5 and 6, we observed a large drop in the ICC, with a high CI95, but no conclusion can be drawn since the size of the samples was very small, respectively, *n* = 8 and *n* = 5. Further studies should be carried out to obtain statistically relevant data.

The limitation of this study lies in two points, the first being the low number of FSP 5 and 6 patients, an issue which will be addressed in the near future. The second point is that the results are specific to an algorithm developed by I-Virtual with particular characteristics, which might not be applicable to other rPPG algorithms. 

The strength of the study is that it is the very first to assess the applicability of the rPPG technique in current practice in such a large and diverse group of patients. Additionally, the tested population is representative of the patients who would benefit from the use of such measurement tools in telemedicine, as it included all demographic characteristics (age, gender, BMI, phototype, in- and outpatients of a large hospital). 

## 5. Conclusions

To our knowledge, this study provides the largest series conducted in patients during real-life clinical settings and is the first to evaluate the accuracy of the rPPG assessment of HR in subjects. We observed robust accuracy of the rPPGc assessment of HR in patients with skin tones from 1 to 4. Further clinical studies are needed to specifically address the limitations of this measurement technique in the darkest skin tones (FSP 5 and 6). Our results pave the way for a greater use of HR in current practice and broaden its measurement in telemedicine. It would also be clinically relevant to clarify the interest of this rPPG method in mobile real-life HR monitoring and, more specifically, during physical activity.

## Figures and Tables

**Table 1:** Baseline demographic and clinical characteristics of included patients (*n* = 963).

Female, *n* (%)	471 (48.9%)
Age, mean (SD), years	56.6 (±16.0)
Body mass index, mean (SD), kg/m^2^	28.1 (±7.3)
BMI < 30, *n* (%)	650 (67.5%)
Class 1 obesity, *n* (%)	172 (17.9%)
Class 2 obesity, *n* (%)	67 (7.0%)
Class 3 obesity, *n* (%)	74 (7.7%)
Fitzpatrick skin colour scale, *n* (%)	
1	20 (2.1%)
2	512 (3.2%)
3	360 (37.4%)
4	58 (6.0%)
5	8 (0.8%)
6	5 (0.5%)

Legend: Data are presented as *n* (%) for dichotomous variables, mean (±SD) for continuous demographic variables with normal distribution and median (interquartile range) with non-normal distribution.

**Table 2:** Intersystem concordance between rPPGc and gold standard as a function of age.

	*n* (%)	ICC	CI95
Ages groups, scale, *n* (%)			
18–29 years	78 (8.1)	0.883	[0.817:0.925]
30–39 years	87 (9.0)	0.804	[0.700:0.872]
40–49 years	127 (13.2)	0.942	[0.917:0.959]
50–59 years	201 (20.9)	0.875	[0.836:0.906]
60–69 years	254 (26.4)	0.842	[0.798:0.876]
70–79 years	163 (16.9)	0.912	[0.880:0.935]
over 80 years	53 (5.5)	0.908	[0.841:0.947]

Legend: CI95, confidence interval 95%; ICC, intraclass correlation coefficient; rPPGc, Caducy remote photoplethysmography imaging.

**Table 3:** Intersystem concordance between rPPGc and gold standard according to body mass index (BMI) and phototype.

	ICC	CI95
Body mass index		
BMI < 30	0.875	[0.854:0.893]
Class 1 obesity	0.955	[0.940:0.967]
Class 2 obesity	0.712	[0.532:0.823]
Class 3 obesity	0.979	[0.966:0.987]
Fitzpatrick skin colour scale		
1	0.927	[0.820:0.971]
2	0.901	[0.882:0.917]
3	0.850	[0.817:0.885]
4	0.981	[0.967:0.988]
5	0.416	[−1.644:0.881]
6	-0.204	[−7.898:0.871]

Legend: CI95, confidence interval 95%; ICC, intraclass correlation coefficient; rPPGc, Caducy remote photoplethysmography imaging.

**Table 4:** Intrasystem correlation between rPPGc as a function of time.

Time	30 s	60 s	120 s
**30 s**			
**60 s**	0.998 [0.998:0.998]		
**120 s**	0.997 [0.996:0.997]	0.999 [0.998:0.999]	

Legend: Data are presented as intraclass correlation coefficient (ICC) with a 95% confidence interval [CI].

## Data Availability

Not applicable.
